# Trehalose Recycling Promotes Energy-Efficient Biosynthesis of the Mycobacterial Cell Envelope

**DOI:** 10.1128/mBio.02801-20

**Published:** 2021-01-19

**Authors:** Amol Arunrao Pohane, Caleb R. Carr, Jaishree Garhyan, Benjamin M. Swarts, M. Sloan Siegrist

**Affiliations:** a Department of Microbiology, University of Massachusetts, Amherst, Massachusetts, USA; b Department of Chemistry and Biochemistry, Central Michigan University, Mount Pleasant, Michigan, USA; c Molecular and Cellular Biology Graduate Program, University of Massachusetts, Amherst, Massachusetts, USA; University of Pittsburgh

**Keywords:** *Mycobacterium*, mycomembrane, oxidative stress, starvation, trehalose

## Abstract

The mycomembrane layer of the mycobacterial cell envelope is a barrier to environmental, immune, and antibiotic insults. There is considerable evidence of mycomembrane plasticity during infection and in response to host-mimicking stresses. Since mycobacteria are resource and energy limited under these conditions, it is likely that remodeling has distinct requirements from those of the well-characterized biosynthetic program that operates during unrestricted growth. Unexpectedly, we found that mycomembrane remodeling in nutrient-starved, nonreplicating mycobacteria includes synthesis in addition to turnover. Mycomembrane synthesis under these conditions occurs along the cell periphery, in contrast to the polar assembly of actively growing cells, and both liberates and relies on the nonmammalian disaccharide trehalose. In the absence of trehalose recycling, *de novo* trehalose synthesis fuels mycomembrane remodeling. However, mycobacteria experience ATP depletion, enhanced respiration, and redox stress, hallmarks of futile cycling and the collateral dysfunction elicited by some bactericidal antibiotics. Inefficient energy metabolism compromises the survival of trehalose recycling mutants in macrophages. Our data suggest that trehalose recycling alleviates the energetic burden of mycomembrane remodeling under stress. Cell envelope recycling pathways are emerging targets for sensitizing resource-limited bacterial pathogens to host and antibiotic pressure.

## Abstract (precis)

The mycomembrane layer of the mycobacterial cell envelope is a barrier to environmental, immune, and antibiotic insults. There is considerable evidence of mycomembrane plasticity during infection and in response to host-mimicking stresses.

## INTRODUCTION

The mycobacterial cell envelope is comprised of covalently bound peptidoglycan, arabinogalactan, and mycolic acids, as well as intercalated glycolipids and a thick capsule (1). The mycolic acids attached to the arabinogalactan and the noncovalent glycolipids, respectively, form the inner and outer leaflets of the mycomembrane, a distinctive outer membrane present in members of the *Corynebacterineae* suborder. The mycomembrane is a key determinant of envelope permeability and home to a variety of immunomodulatory lipids and glycolipids (2–4). There is substantial evidence that the mycomembrane is remodeled *in vivo* and in response to host-mimicking stresses, conditions in which mycobacterial growth and envelope synthesis are presumed to be slow or nonexistent (3, 5–13). While these studies have elucidated bulk changes in mycomembrane composition, the dynamics and subcellular distribution of the molecular transitions have not been characterized. It is also unclear in most cases whether the alterations are solely catabolic, or whether anabolic reactions also contribute to changes in mycomembrane composition under stress.

**FIG 1:**
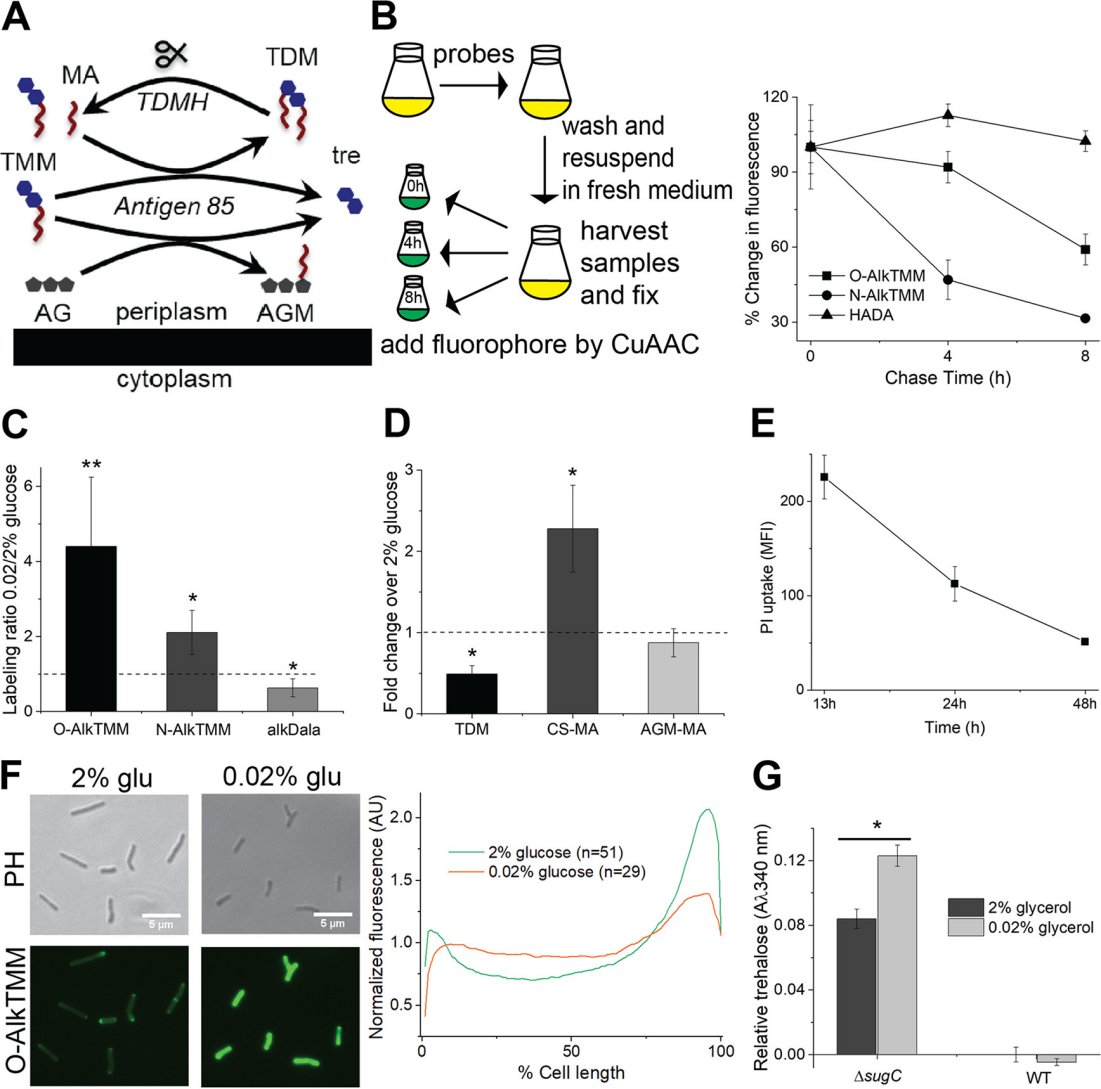
Mycomembrane synthesis and degradation are active under carbon limitation. (A) Mycomembrane synthesis and degradation. TMM, trehalose monomycolate; TDM, trehalose dimycolate; AG, arabinogalactan; AGM, arabinogalactan mycolates; MA, free mycolic acids; TDMH, TDM hydrolase. (B) TDM turnover under nutrient deprivation. M. smegmatis was cultured in 0.02% glucose-supplemented medium in the presence of metabolic probes O-AlkTMM (primarily labels AGM), N-AlkTMM (labels TDM), or HADA (labels cell wall peptidoglycan). After 24 h, the cultures were washed and resuspended in probe-free medium. Aliquots were removed 0, 4, and 8 h into the chase and fixed with 2% formaldehyde. Alkynes were detected by copper-catalyzed azide-alkyne cycloaddition (CuAAC) reaction with carboxyrhodamine-110 azide. Fluorescence was quantitated by flow cytometry, with the median fluorescence intensities (MFIs) were normalized to the initial, 0-h time point for each probe. The experiment was performed three times in triplicate; the results of one representative experiment are shown. (C) Metabolic labeling of M. smegmatis in 0.02% glucose-supplemented medium with O-AlkTMM, N-AlkTMM, and alkDala (labels peptidoglycan). Alkynes were detected by CuAAC reaction with carboxyrhodamine-110 azide. Data were normalized to labeling in 2% glucose-supplemented medium and plotted from four independent experiments. (D) Quantitation of TLC of different mycomembrane components for M. smegmatis in 0.02% glucose-supplemented medium. TDM, trehalose dimycolate; CS-MA, free, culture supernatant mycolic acids; AGM-MA, mycolic acids released from arabinogalactan. TLC results were scanned and processed in ImageJ (99). The data are normalized to TLC results from samples taken from M. smegmatis cultured in 2% glucose-supplemented medium and plotted from three independent experiments. (For representative TLC results, see Fig. S2.) (E) PI staining of M. smegmatis during adaptation to low carbon. M. smegmatis was cultured in 0.02% glucose-supplemented medium. Aliquots were removed at 13, 24, and 48 h and incubated with PI. Fluorescence was quantitated by flow cytometry, and the MFI was plotted. The experiment was performed three times in triplicate; the results of a representative experiment are shown. (F) O-AlkTMM labeling of M. smegmatis AGM in 2 or 0.02% glucose-supplemented medium. Alkynes were detected by CuAAC reaction with carboxyrhodamine-110 azide. (Left) Fluorescence microscopy. Scale bars, 5 μm. (Right) The cellular fluorescence was quantitated for cells lacking visible septa from three independent experiments. The signal was normalized to both cell length and total fluorescence intensity. Cells were oriented such that the brighter pole is on the right-hand side of the graph. A.U., arbitrary units. (G) Quantification of trehalose from supernatants of M. smegmatis wild-type and Δ*sugC* strains cultured in 2 or 0.02% glycerol-supplemented medium. The experiment was performed at least three times in triplicate; the results of one representative experiment are shown. Error bars, standard deviations. The statistical significance of 0.02% versus 2% glucose or glycerol samples from three independent experiments was assessed by two-tailed Student *t* test. *, *P* < 0.05; **, *P* < 0.005.

Recycling pathways are likely to be at the nexus of stress-triggered mycomembrane reorganization. Mycolic acids are ligated to the nonmammalian disaccharide trehalose in the cytoplasm (14). Once transported to the periplasm, trehalose monomycolate (TMM) donates its mycolic acid to arabinogalactan, forming arabinogalactan mycolates (AGM), or to an acceptor TMM, forming trehalose dimycolate (TDM; Fig. 1A). Both processes release free trehalose. TDM can also be degraded by TDM hydrolase (TDMH) into TMM and free mycolic acids, the latter of which are an important component of biofilm extracellular matrix in mycobacteria (7, 15). While a salvage mechanism for mycolic acids is still under debate (16–19), recapture of trehalose occurs via the LpqY-SugABC transporter (20). Depending on the specific environmental demand, mycobacteria may funnel reclaimed trehalose back to central carbon metabolism to generate intermediates for glycolysis or the pentose phosphate pathway or to store it in the cytoplasm, possibly as a stress protectant or compatible solute (6, 21–23). An additional but unexplored potential fate for recaptured trehalose is direct reincorporation into TMM or other glycoconjugates destined for the cell surface. Thus, trehalose connects mycomembrane synthesis and turnover to the metabolic status of the mycobacterial cell.

We find that mycomembrane remodeling triggered by nutrient limitation comprises both synthesis and degradation of AGM and TDM. Remodeling continues in the absence of trehalose recycling. However, compensatory anabolism upsets the energy and redox balance of the cell in a manner indicative of futile cycling (24–28). Similar dysfunction has been proposed to enhance the efficacy of certain antibiotics (29, 30), and indeed, loss of LpqY sensitizes Mycobacterium tuberculosis to multiple drugs (31). M. tuberculosis Δ*sugC* and Δ*lpqY* strains are also known to be attenuated during infection (20, 32, 33). We show here that inefficient ATP metabolism is the primary mechanism of attenuation in macrophages.

While previous studies identified multiple phenotypes for trehalose recycling mutants, they did not explain how the LpqY-SugABC system contributes to mycobacterial fitness. Our data indicate that trehalose recycling minimizes energy consumption and oxidative stress during mycomembrane adaptation to nutrient limitation. Given the energetic costs associated with *de novo* biosynthesis, recycling pathways for trehalose and other mycomembrane components may be particularly important for M. tuberculosis resilience to stress.

## RESULTS

### Mycomembrane synthesis and degradation are active under carbon limitation.

Decreased TDM abundance has been reported for mycobacteria growing in biofilms or adapting to hypoxia or nutrient limitation (3, 5, 7, 23). Since uncontrolled TDM hydrolysis results in cell lysis (7, 34), we sought to understand the kinetics of TDM turnover under stress. TMM donates mycolic acids to other molecules of TMM, to form the TDM glycolipid, or to arabinogalactan, to form covalent arabinogalactan mycolates (AGM, Fig. 1A). The TMM-mimicking probe N-AlkTMM specifically incorporates into TDM because the amide linkage permits mycolic acid acceptance but not donation of the alkyne-appended lipid chain (35). To track TDM hydrolysis under carbon limitation, we performed a pulse-chase experiment in which we labeled M. smegmatis with N-AlkTMM for 12 h in low (0.02%)-glucose-supplemented 7H9 medium then washed the sample before transferring it to 7H9 lacking both the probe and glucose (Fig. 1B, left). Alkyne-labeled TDM was detected on fixed cells at 0, 4, and 8 h posttransfer by copper-catalyzed azide-alkyne cycloaddition (CuAAC) with a fluorescent azide label. We found that TDM labeling decreased by ∼3-fold in this time period (Fig. 1B, right). Fluorescence derived from d-amino acid-labeled cell wall peptidoglycan remained steady, however, consistent with limited bacterial growth under this condition (Fig. 1B, right; see also Fig. S1A in the supplemental material).

10.1128/mBio.02801-20.1 FIG S1 Effect(s) of carbon source and amount on M. smegmatis growth and cell envelope labeling. (A) Growth of M. smegmatis grown in different carbon sources. Each experiment was performed twice in triplicate with similar results; one experiment is shown. The arrow indicates the time at which cultures were labeled with O-AlkTMM or HADA. (B and C) O-AlkTMM (primarily incorporates into AGM) (B) and HADA (incorporates into peptidoglycan) (C) labeling of M. smegmatis cultured in medium supplemented with different carbon sources. Alkynes were detected by CuAAC reaction with carboxyrhodamine-110 azide. The data are normalized to labeling in 2% glucose-supplemented medium and plotted from three independent experiments. Cyan autofluorescence subtracted from values in panel C. Error bars, standard deviations. Download FIG S1, TIF file, 0.6 MB. Copyright © 2021 Pohane et al. 2021 Pohane et al. This content is distributed under the terms of the Creative Commons Attribution 4.0 International license.

Under acid stress, nonreplicating but metabolically active M. tuberculosis make new TDM (9). We found that N-AlkTMM uptake (no chase) increased ∼2-fold in low-glucose medium (Fig. 1C). However, a decline in the steady-state abundance of TDM (Fig. 1D; see also Fig. S2B) suggested that enhanced synthesis is outweighed by the TDM turnover observed in the pulse-chase experiment (Fig. 1B, right).

10.1128/mBio.02801-20.2 FIG S2 Quantitation of mycomembrane components in high and low carbon media. (A) Mycomembrane components extracted from M. smegmatis and analyzed by TLC. TDM, trehalose dimycolate; AGM-MA, covalently bound mycolic acids; CS-MA, free mycolic acids from culture supernatant. (B to D) Representative TLCs of TDM (B), AGM-MA (C), and CS-MA (D). Wild-type (WT) and Δ*sugC* M. smegmatis strains were cultured for 24 h in 0.02 or 2% glucose-supplemented medium and processed for lipid extraction. Samples were normalized by wet pellet weight (B), by the extracted mAGP dry weight (C), or by optical density (D). The black boxes highlight the bands used for quantification (Fig. 1D), and arrows denote the standards. STD, standard (purified TDM or mycolic acids). Download FIG S2, TIF file, 1.1 MB. Copyright © 2021 Pohane et al. 2021 Pohane et al. This content is distributed under the terms of the Creative Commons Attribution 4.0 International license.

We hypothesized that there were additional changes in mycomembrane metabolism. O-AlkTMM is also a TMM-mimicking probe but features an ester-linked lipid chain. While the molecule can serve as either an alkyne-lipid donor or acceptor, ∼90% of labeling from this probe is present in the M. smegmatis AGM cellular fraction (35). O-AlkTMM uptake was enhanced in low-glucose medium to a greater extent than N-AlkTMM (Fig. 1C). The fluorescence signal derived from this probe was also more persistent than N-AlkTMM in a no-probe, no-glucose chase (Fig. 1B).

A variety of carbohydrates can serve as mycolate acceptors, including glucose (36, 37). High levels of glucose in the growth medium might therefore suppress O-AlkTMM labeling of the cell surface by competing with arabinogalactan. While in our labeling window M. smegmatis grew faster in 7H9 medium with high (2%) versus medium (0.2%) glucose supplementation, O-AlkTMM-derived fluorescence in the high-glucose condition was lower (see Fig. S1B). However, O-AlkTMM labeling was similar for M. smegmatis in 0.2 or 0.02% glucose or acetate (see Fig. S1B), despite sluggish or absent bacterial replication under the low carbon conditions (see Fig. S1A). Thus, incorporation of O-AlkTMM into AGM is suppressed in high glucose, likely because the alkyne-fatty acid from the probe is transferred to the unanchored glucose and washed away. Nonetheless our data indicate that substantial AGM synthesis occurs in growth-limiting amounts of glucose or acetate. Since the steady-state abundance of the molecule did not change in carbon-limited medium (Fig. 1D; see also Fig. S2C), these experiments also suggest that AGM synthesis is balanced by the turnover that we observed by pulse-chase (Fig. 1B, right).

We previously showed that the fluorescent d-amino acid HADA as well as alkyne-d-alanine (alkDala) incorporate into M. smegmatis peptidoglycan via both cytoplasmic and l,d-transpeptidase enzymes (38). HADA and alkDala labeling roughly correlated with mycobacterial growth rate under different amounts of glucose or acetate (Fig. 1C; see also Fig. S1A and C in the supplemental material). Suppressed levels of peptidoglycan synthesis or remodeling during carbon limitation stood in contrast to active mycomembrane metabolism.

### AGM synthesis occurs along the periphery of the mycobacterial cell during carbon limitation.

TDM hydrolysis enhances envelope permeability in oleic acid- and glucose-deprived M. tuberculosis (3). Surprisingly, despite an analogous decrease in TDM abundance (Fig. 1D; see also Fig. S2B), M. smegmatis became less permeable to propidium iodide when cultured in glucose-limited medium (Fig. 1E). Global AGM levels have also been linked to mycobacterial permeability (39). Although AGM abundance was relatively unaffected in glucose-deprived medium (Fig. 1D; see also Fig. S2C), our data suggest that the apparent stasis belies active synthesis and degradation (Fig. 1B and C). We considered whether AGM remodeling might impact its spatial distribution, which in turn could alter cell permeability.

Mycobacteria growing in nutrient-replete medium construct their cell envelope in gradients that emanate from the poles and continue along the sidewall (35, 38, 40–48). While polar peptidoglycan synthesis promotes cell elongation, sidewall synthesis occurs in response to cell wall damage (38). We hypothesized that the AGM synthesis that we observe under carbon deprivation (Fig. 1C) is a cell-wide response, similar to peptidoglycan repair. Quantitative fluorescence microscopy revealed that O-AlkTMM labeling of M. smegmatis growing in carbon-replete medium comprised polar gradients (Fig. 1F) as expected (35, 38). However, in slow- or nongrowing, carbon-deprived M. smegmatis, O-AlkTMM-labeled species were more evenly distributed around the periphery of the cell. This observation suggests that AGM synthesis fortifies the mycomembrane along the sidewall as mycobacteria adapt to carbon deprivation.

### Trehalose cycling supports mycomembrane metabolism during carbon starvation.

Mycomembrane synthesis centers on the mycolic acid donor trehalose monomycolate (TMM). Prior to its export to the periplasm, TMM is synthesized in the cytoplasm by the ligation of a mycolic acid to trehalose (50). *De novo* synthesis of mycolic acids and trehalose is both energy and resource intensive; recycling pathways for both molecules have been shown or proposed (18–20). We hypothesized that nutrient-starved mycobacteria might buffer the costs of TMM synthesis by enlisting recycling pathways. Since the recycling mechanism for mycolic acids is still controversial (16, 17), we focused on the role of trehalose uptake.

**FIG 2:**
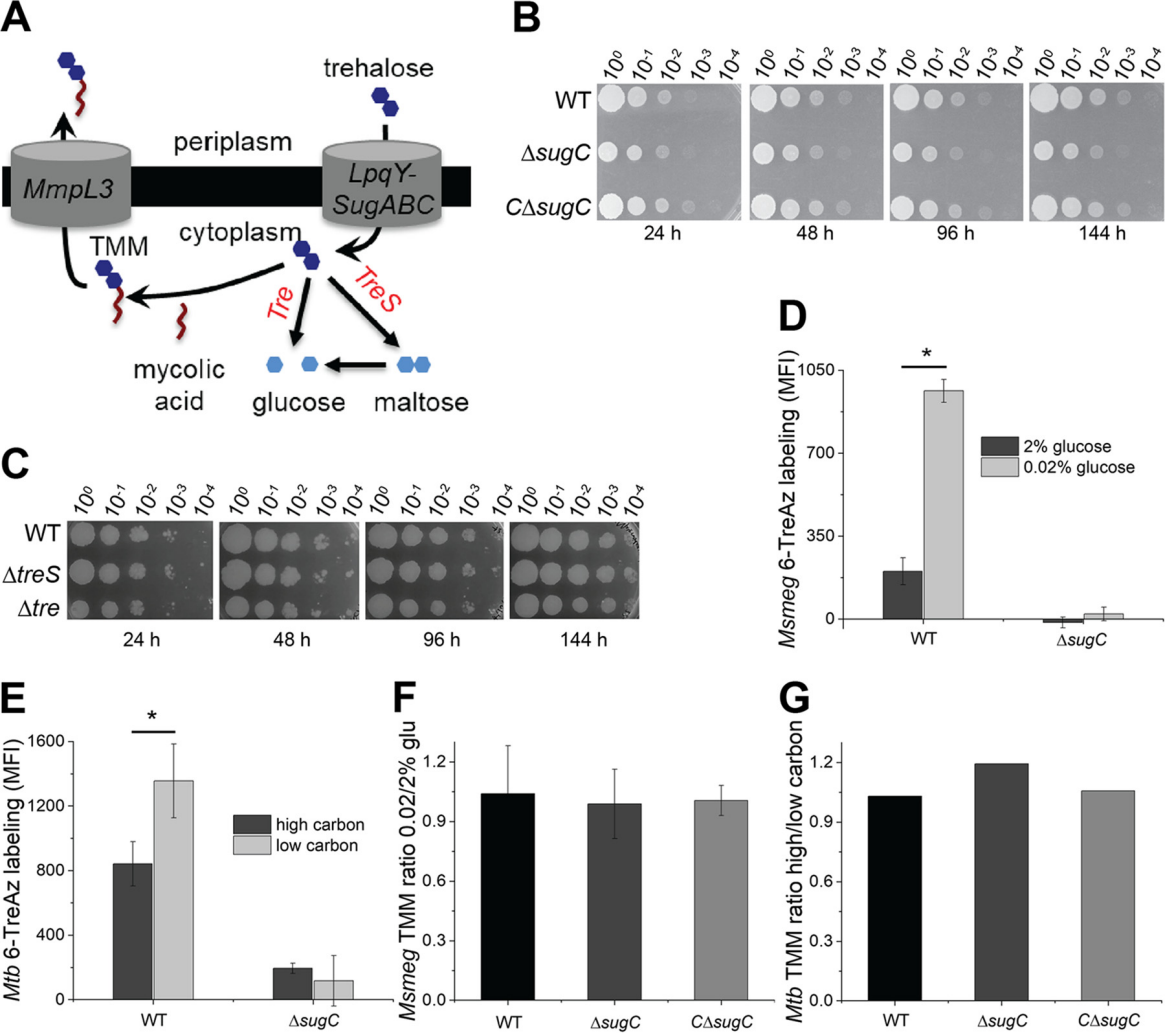
Trehalose cycling supports mycomembrane metabolism during carbon limitation. (A) Potential fates of recycled trehalose in catabolism (trehalase [Tre] or TreS) or in trehalose monomycoate (TMM) biosynthesis. (B and C) Survival of wild-type, Δ*sugC*, complemented Δ*sugC* (CΔ*sugC*), Δ*treS*, and Δ*tre* M. smegmatis strains in 0.02% glucose-supplemented medium. Tenfold serial dilutions were plated at the indicated time points. The experiment was performed two times with similar results; the results of one experiment are shown. (D and E) 6-TreAz labeling of wild-type and Δ*sugC* M. smegmatis (*Msmeg*) and M. tuberculosis (*Mtb*) cultured in low- or high-carbon medium. Azides were detected by strain-promoted azide-alkyne cycloaddition (SPAAC) with DBCO-Cy5 label. The fluorescence was detected by flow cytometry, with MFI values from controls lacking 6-TreAz (but subjected to SPAAC) subtracted from the sample MFI. The experiment was performed at least three times in triplicate; the results of one representative experiment are shown. (F and G) TMM abundance in M. smegmatis and M. tuberculosis cultured in low- or high-carbon medium. TLC results were scanned and processed in ImageJ (99). The data are normalized to the TLC results from mycobacteria cultured in high-carbon medium and plotted from two (M. tuberculosis) or three (M. smegmatis) independent experiments. (For representative TLC results, see Fig. S3B and C.) Error bars, standard deviations. The statistical significance of low- versus high-carbon samples was assessed by two-tailed Student *t* test. *, *P* < 0.05.

Trehalose released as a by-product of extracellular mycomembrane metabolism is recycled via the LpqY-SugABC transporter (20) (Fig. 2A). At least two different processes liberate trehalose: (i) ligation of mycolic acids from TMM to arabinogalactan to form AGM and (ii) transfer of mycolic acids from TMM to another molecule of TMM to form TDM (Fig. 1A). Breakdown of TDM by the TDM hydrolase (TDMH) yields TMM and mycolic acids (7, 15, 34), so subsequent use of TMM in the foregoing reactions would also release trehalose. Our metabolic labeling results suggested that all of these processes are active as M. smegmatis adapts to carbon limitation (Fig. 1). We were unable to measure extracellular trehalose levels in wild-type M. smegmatis, presumably because LpqY-SugABC rapidly internalizes the disaccharide (20). However, by using M. smegmatis Δ*sugC*, a strain that lacks a functional trehalose transporter, we were able to detect elevated levels of trehalose in the supernatant when bacteria were grown in carbon-limited conditions (Fig. 1G; note that we used glycerol as the carbon source as glucose interferes with the assay). We also found that free mycolic acids accumulated in the supernatant of low glucose cultures (Fig. 1D; see also Fig. S2D), as expected from TDM turnover. Together, our data indicate that trehalose is liberated upon reorganization of the mycomembrane.

10.1128/mBio.02801-20.3 FIG S3 Metabolic labeling and quantitation of TMM in high- and low-carbon media. (A) Metabolic labeling of wild-type and Δ*sugC* M. smegmatis strains in 0.02 and 2% glucose-supplemented medium with 6-TreAz (labels TMM). After fixation, alkynes were detected by CuAAC reaction with carboxyrhodamine-110 alkyne. Scale bars, 10 µm. (B and C) Representative TLC images of TMM from wild-type (WT), Δ*sugC*, and complemented (*C*Δ*sugC*) M. smegmatis (B) strains or M. tuberculosis (C) cultured for 24 h in low- or high-carbon medium. Samples were normalized by wet pellet weight in panel B or by optical density in panel C. The black boxes highlight the bands used for quantification (Fig. 2F and G), and arrows denote the standards. STD, standard (purified TMM). For panel C, a cropped image with enhanced contrast is included below the main image to highlight the TMM standard. Download FIG S3, TIF file, 1.3 MB. Copyright © 2021 Pohane et al. 2021 Pohane et al. This content is distributed under the terms of the Creative Commons Attribution 4.0 International license.

Exogenously supplied trehalose can support mycobacterial growth (20) after it is transported by LpqY-SugABC (20) and metabolized by trehalase (21) or TreS (6, 50–52) (Fig. 2A). We recovered similar CFU for Δ*sugC*, Δ*tre*, Δ*treS*, and wild-type M. smegmatis strains from 1, 2, 4, and 6 days in low glucose (Fig. 2B and C). These data suggest that trehalose catabolism is not required for viability, nor does it fuel appreciable cell growth, under carbon deprivation. Given that both the optical density and CFU of M. smegmatis were steady (Fig. 2B and C; see also Fig. S1A), trehalose recovered from the mycomembrane also does not fuel appreciable cell growth under this condition.

In hypoxic and biofilm cultures of M. tuberculosis, TMM and TDM levels decrease (5, 6, 23). Glycolipid turnover occurs rapidly in the former, within 4 h (6), and slowly in the latter, within 16 days (23). We did not observe a net decrease in TMM for M. smegmatis or M. tuberculosis under carbon limitation (Fig. 2F and G) despite an increase in TMM-consuming AGM and TDM remodeling (Fig. 1C). We posited that TMM pools might be replenished by recycled trehalose. Metabolic incorporation of exogenous 6-azido-trehalose (6-TreAz) by M. smegmatis or M. bovis BCG requires uptake by LpqY-SugABC (53). We found that 6-TreAz labeling was enhanced in slow-growing, glucose-starved M. smegmatis (Fig. 2D) or oleic acid- and glucose-starved M. tuberculosis (Fig. 2E) (3). As incorporation of the metabolite was respectively abolished or diminished in Δ*sugC* M. smegmatis (Fig. 2D; see also Fig. S3A) (53) or M. tuberculosis (Fig. 2E), enhanced 6-TreAz labeling under carbon limitation indicates an increase in trehalose recycling.

6-TreAz recovered by the LpqY-SugABC transporter may remain intact in the cytoplasm, be catabolized, or be converted to azido-TMM and transported outside the cell (Fig. 2A) (53). Although it has not been reported, it is possible that the probe incorporates into other trehalose-bearing molecules in the mycobacterial envelope (21). To tune our detection for the cell surface, we selected DBCO-Cy5 as the fluorescent, azide-reactive label because the localized charge on the sulfonated cyanine dye confers poor membrane permeability (54). The enhanced 6-TreAz labeling that we observed for M. smegmatis and M. tuberculosis during carbon limitation (Fig. 2D and E) strongly suggests that at least some of the recycled trehalose is converted into an envelope component(s). Given that (i) TMM and TDM are the only known trehalose-containing glycoconjugates shared by both M. smegmatis and M. tuberculosis and that (ii) TDM cannot be labeled by 6-TreAz (53), we conclude that TMM is the most likely target. As steady-state TMM levels remained relatively constant in both species (Fig. 2F and G; see Fig. S3B and C), enhanced conversion of 6-TreAz to azido-TMM further suggests that trehalose recycling under carbon deprivation helps to maintain TMM levels. These data are consistent with a model in which trehalose cycles in and out of the cell to remodel the mycomembrane in carbon-deprived mycobacteria.

### Mycomembrane reorganization under carbon deprivation can occur in the absence of trehalose cycling.

Our experiments suggest that trehalose cycling contributes to mycomembrane reorganization during carbon limitation. However, loss of trehalose import by LpqY-SugABC did not impact the abundance of TMM, TDM or AGM (see Fig. S2B, S2C, S3B, S3C, S4B, and S4C); synthesis of AGM or TDM (see Fig. S4D); turnover of TDM (compare Fig. 1B, right, to Fig. S4E); or permeability (see Fig. S4F). The absence of measurable changes in mycomembrane metabolism or composition were consistent with earlier work showing that M. tuberculosis Δ*sugC* and Δ*lpqY* strains do not have detectable changes in the glycolipid composition of their mycomembranes compared to wild type (20). These data also indicate that mycomembrane reorganization can occur in the absence of trehalose recycling.

10.1128/mBio.02801-20.4 FIG S4 Mycomembrane reorganization under carbon deprivation does not require trehalose recycling. (A) Mycomembrane biosynthesis and turnover continues in the absence of trehalose recycling. Quantitation of TMM abundance in Δ*sugC* and complemented (CΔ*sugC*) M. smegmatis strains (B) or M. tuberculosis (C) cultured for 24 h in low-carbon medium. TLCs were scanned and processed in ImageJ (99). Data were normalized to wild-type M. smegmatis (A) or M. tuberculosis (B) and plotted from three independent experiments, including images from Fig. S3B and C. (D) Quantitation of metabolic labeling of wild-type and Δ*sugC* M. smegmatis in 0.02 and 2% glucose-supplemented medium with O-AlkTMM (primarily labels AGM), N-AlkTMM (labels TDM), and alkDala (labels peptidoglycan). After fixation, alkynes were detected by CuAAC reaction with carboxyrhodamine 110 azide. Fluorescence was quantitated by flow cytometry and is expressed as the MFI. The experiment was performed three times in triplicate; one representative experiment is shown. (E) TDM turnover in Δ*sugC* M. smegmatis under carbon deprivation (compare to data for wild-type in Fig. 1B, right). M. smegmatis Δ*sugC* was cultured in 0.02% glucose-supplemented medium in the presence of metabolic probes O-AlkTMM (primarily labels AGM), N-AlkTMM (labels TDM), or HADA (labels cell wall peptidoglycan). After 24 h, the cultures were washed and resuspended in probe-free medium. Aliquots were removed 0, 4, and 8 h into the chase and fixed with 2% formaldehyde. Alkynes were detected by copper-catalyzed azide-alkyne cycloaddition (CuAAC) reaction with carboxyrhodamine-110 azide. Fluorescence was quantitated by flow cytometry, with the MFIs normalized to the initial, 0-h time point for each probe. The experiment was performed three times in triplicate; one representative experiment is shown. (F) PI staining of wild-type and Δ*sugC* M. smegmatis strains after 24 h in 0.02% glucose-supplemented medium. Fluorescence was quantitated by flow cytometry, and the MFI was plotted. The experiment was performed three times in triplicate; one representative experiment is shown. Error bars, standard deviations. Download FIG S4, TIF file, 1.2 MB. Copyright © 2021 Pohane et al. 2021 Pohane et al. This content is distributed under the terms of the Creative Commons Attribution 4.0 International license.

### Trehalose recycling promotes redox and energy homeostasis under carbon limitation.

While trehalose recycling was dispensable for M. smegmatis and M. tuberculosis mycomembrane remodeling and survival under carbon limitation, we hypothesized that it might be important for withstanding other stressors. We first sought to determine whether blocking trehalose recycling disrupts redox homeostasis. We tested this hypothesis under growth-limiting (see Fig. S1) (3) carbon limitation since trehalose recycling is enhanced under this condition (Fig. 2D and E).

**FIG 3:**
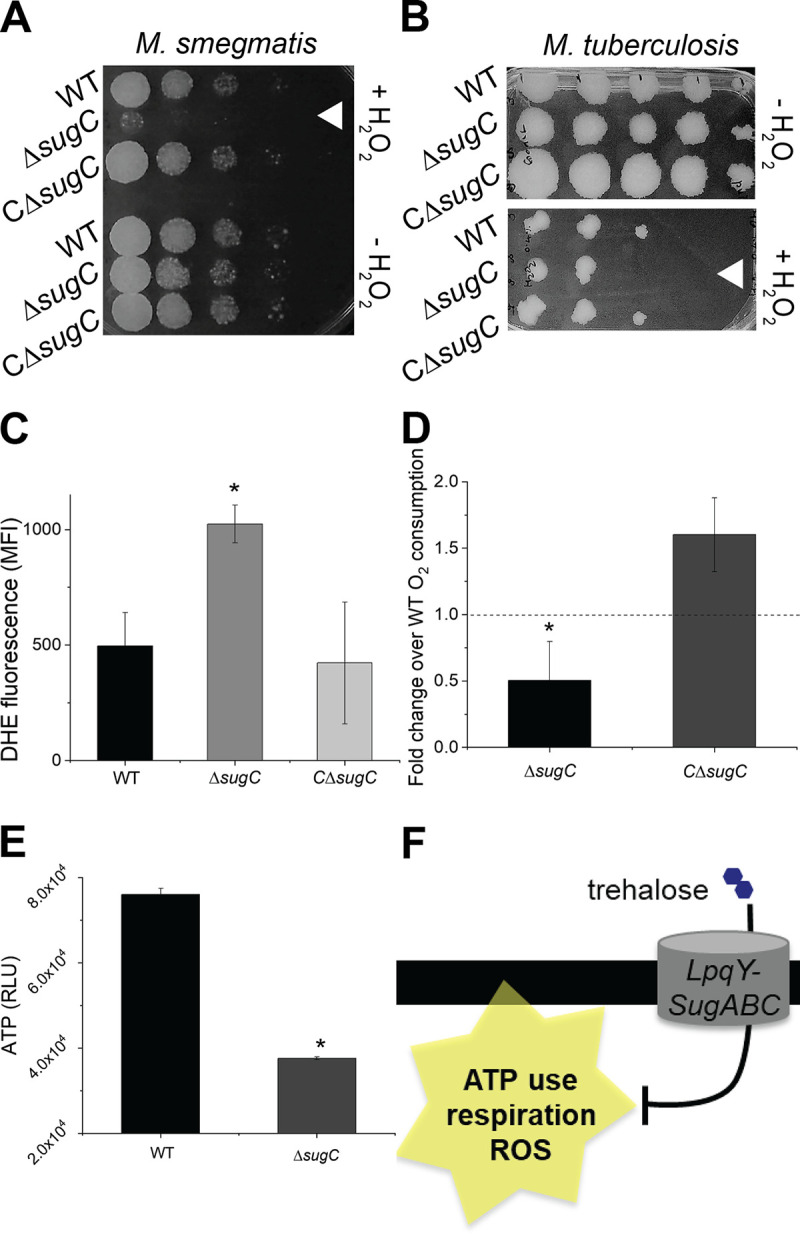
Trehalose recycling promotes redox and energy homeostasis under carbon limitation. (A and B) Sensitivity of carbon-deprived wild-type, Δ*sugC*, and complemented Δ*sugC* (CΔ*sugC*) M. smegmatis (A) or M. tuberculosis (B) strains to hydrogen peroxide. Tenfold serial dilutions were plated. White triangles highlight the most sensitive strain or condition. The sensitivity of each strain or condition was assessed at least three independent times; representative data are shown. (C) Staining of M. smegmatis cultured in 0.02% glucose-supplemented medium by superoxide indicator dye dihydroethidium (DHE). Fluorescence was detected by flow cytometry, and the MFI was plotted. The experiment was performed three times in triplicate; the results of one representative experiment are shown. (D) Oxygen consumption of M. smegmatis cultured in 0.02% glucose-supplemented medium. Strains were incubated with or without methylene blue, and the absorbance at 665 nm was measured. The absorbance from untreated samples was subtracted and then values were normalized to those of the wild-type. The data are plotted for three independent experiments performed in triplicate. (E) ATP levels of M. smegmatis cultured in 0.02% glucose-supplemented medium. Protein concentration-normalized cell lysates were incubated with BacTiter-Glo reagent, and the luminescence was measured in relative light-forming units (RLU). The experiment was performed at least three times in triplicate; the results of one representative experiment are shown. (F) Cartoon summary of Fig. 3 and Fig. S5. Error bars, standard deviation. For panels C to E, the statistical significance of Δ*sugC* or complement strains versus the wild type from at least three independent experiments was assessed by a two-tailed Student *t* test. *, *P* < 0.05.

M. smegmatis and M. tuberculosis Δ*sugC* strains were sensitized to exogenously applied hydrogen peroxide and/or to reactive oxygen species (ROS)-potentiating vitamin C (55) (Fig. 3A and B; see also Fig. S5A and B). Loss of trehalose recycling also enhanced the fluorescence of dihydroethidium (DHE), an indicator dye of endogenous cellular superoxide (Fig. 3C) (56). Propidium iodide staining remained unchanged (see Fig. S4F), suggesting that the effect was not due to nonspecific differences in uptake, efflux, or cell size. In M. smegmatis, the total pool of cytoplasmic thiol antioxidants was modestly enhanced in the absence of *sugC* (see Fig. S5C). We hypothesized that the increase in free thiols in the *sugC* mutant might be an adaptation to counteract the higher basal levels of superoxide. Consistent with a drive to maintain a reduced thiol pool (57) (58), we observed increased NADP:NADPH (see Fig. S5D) in M. smegmatis Δ*sugC.* Taken together, our data suggest that trehalose recycling that occurs during carbon limitation supports redox balance.

10.1128/mBio.02801-20.5 FIG S5 Trehalose recycling promotes redox balance under carbon limitation. (A) Effect of thiourea pretreatment on hydrogen peroxide sensitivity of carbon-deprived Δ*sugC* M. smegmatis. (B) Sensitivity of carbon-deprived wild-type, Δ*sugC*, and complemented Δ*sugC* (CΔ*sugC*) M. tuberculosis strains to vitamin C. For both panels A and B, 10-fold serial dilutions were plated. White triangles highlight the most sensitive strain or condition. The experiments were performed at least three times; representative data are shown. (C) Quantification of total free thiols. Cell lysates of M. smegmatis strains that had been cultured in 0.02% glucose-supplemented medium were incubated with 5,5′-dithiobis (2-nitrobenzoic acid), and the absorbance at 412 nm was measured. The experiment was performed two times in triplicate with similar results; the results of one experiment are shown. (D) NADP^+^/NADPH levels from M. smegmatis cultured in 0.02% glucose-supplemented medium. Ratios plotted from three independent experiments performed in triplicate. The statistical significance of Δ*sugC* versus wild-type NADP/NADPH ratios was assessed by a two-tailed Student *t* test. **, *P* < 0.005. Download FIG S5, TIF file, 2.4 MB. Copyright © 2021 Pohane et al. 2021 Pohane et al. This content is distributed under the terms of the Creative Commons Attribution 4.0 International license.

A possible endogenous source of ROS in the bacterial cell is respiration, which in turn can be estimated by the oxidation of the methylene blue dye (59). In carbon-limited medium, we observed more methylene blue decolorization for the Δ*sugC* mutant (Fig. 3D), indicating that respiration is enhanced in the absence of trehalose recycling. Notably, however, the mutant had lower levels of ATP than the wild type (Fig. 3E). These data are consistent with a model in which trehalose recycling maintains redox balance in carbon-limited mycobacteria by minimizing ATP consumption and respiration (Fig. 3F). Alternatively, or additionally, redox balance may enable energy homeostasis under this condition.

### Trehalose anabolism disrupts redox balance under carbon limitation.

**FIG 4:**
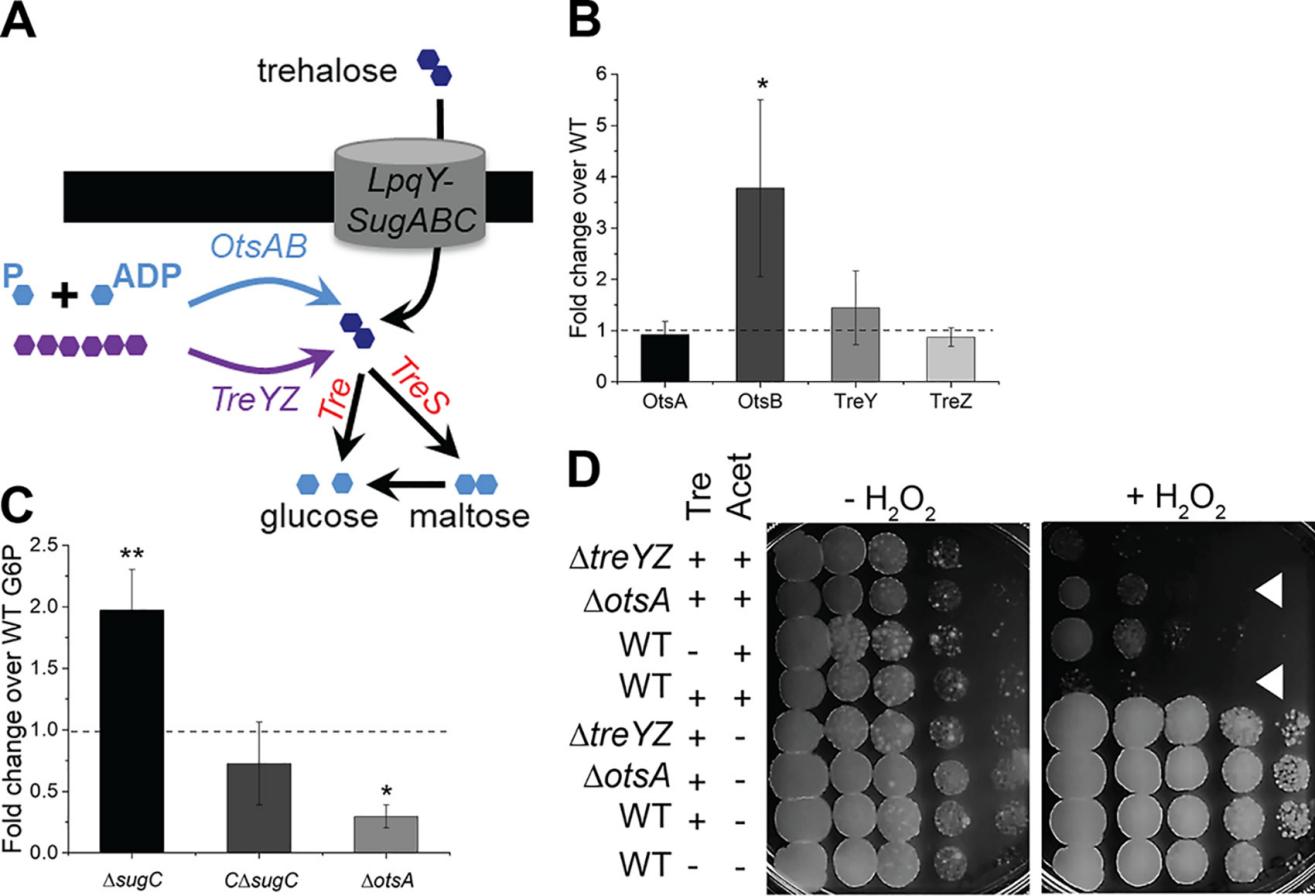
Trehalose anabolism disrupts redox balance under carbon limitation. (A) Anabolic and catabolic pathways for trehalose. Light blue, phosphorylated glucose intermediates; purple, α-glucan polymer. (B) Expression of trehalose biosynthesis genes by qRT-PCR. Wild-type and Δ*sugC* M. smegmatis strains were cultured in 0.02% glucose-supplemented medium. Expression data were first normalized to the housekeeping gene *sigA* and then plotted as a ratio of the Δ*sugC* mutant to the wild type. The data are combined from three independent experiments performed in triplicate. (C) Glucose-6-phosphate (G6P) levels of M. smegmatis cultured in 0.02% glucose-supplemented medium. Protein concentration-normalized cell lysates were incubated with G6P working solution, and the G6P level was measured in a 96-well plate by monitoring the absorbance ratio at 575 nm/605 nm. The data are plotted for three independent experiments performed in duplicate. G6P levels normalized to those of the wild type. (D) Sensitivity of carbon-deprived M. smegmatis to hydrogen peroxide upon trehalase overexpression. Tenfold serial dilutions were plated at the indicated time points. White triangles highlight the difference in sensitivity with or without *otsA*. –Tre, plasmid backbone only; +Tre, plasmid with gene encoding trehalase under acetamide-inducible promoter; Acet, acetamide. The sensitivity of each strain or condition was assessed at least three independent times; representative data shown. Error bars, standard deviations. The statistical significance of expression in the Δ*sugC* mutant relative to the wild-type (B) or of other strains versus the wild type (C) was assessed by two-tailed Student *t* test. *, *P* < 0.05; **, *P* < 0.005.

Cytoplasmic trehalose can protect against ROS directly, in plants, fungi, and other bacteria (60–63), or indirectly, via TreS-dependent catabolism in mature M. tuberculosis biofilms (23). To test whether either of these potential mechanisms could account for recycling-promoted redox homeostasis, we measured the total trehalose pools, endogenous ROS levels, and exogenous ROS sensitivity of mutants defective in trehalose catabolism or anabolism. There are several metabolic pathways for trehalose in mycobacteria: OtsA and OtsB convert phosphorylated glucose intermediates to trehalose; TreY and TreZ degrade the glucose polymer α-glucan into trehalose; TreS converts trehalose to maltose; trehalase degrades trehalose into glucose (Fig. 2A and 4A; see also Fig. S6A). We found that changes to the size of the trehalose pool that were due to perturbations in catabolism (see Fig. S6G and H) or anabolism (see Fig. S6B) did not correlate with endogenous ROS levels (see Fig. S6C) or sensitivity to exogenous ROS (see Fig. S6D, E, and F). These experiments indicated that the mycobacterial redox balance does not depend solely on the size of the trehalose pool or on trehalose catabolism during short-term carbon limitation.

10.1128/mBio.02801-20.6 FIG S6 Depletion of trehalose pool or inhibition of trehalose catabolism is not sufficient to cause oxidative stress. (A) Trehalose biosynthetic pathways. Light blue, phosphorylated glucose intermediates; purple, α-glucan polymer. (B) Quantification of intracellular trehalose from M. smegmatis cultured in 0.02% glucose-supplemented medium. Data are plotted from three independent experiments performed in triplicate. The obtained values for colorimetric detection of trehalose were normalized to the wild type. (C) Staining of M. smegmatis cultured in 0.02% glucose-supplemented medium by the superoxide indicator dye dihydroethidium (DHE). Fluorescence was detected by flow cytometry, and MFIs of the different mutants were normalized to the wild type. Data are plotted from two independent experiments for the complemented Δ*sugC* (CΔ*sugC*) strain and from four independent experiments for the rest of the strains. The experiments were performed in triplicate. The DHE fluorescence for all strains was normalized to that of the wild-type strain. (D to F) Hydrogen peroxide sensitivity of carbon-deprived wild-type and mutant M. smegmatis strains. Tenfold serial dilutions were plated at the indicated time points. Experiments were performed two to three times; representative data are shown. White triangles highlight the most sensitive strains or conditions. –Tre, plasmid backbone pYAB-EV only; +Tre, plasmid with gene encoding trehalase (pYAB-Tre) under acetamide-inducible promoter; Acet, acetamide. Experiments were performed two to three times; representative data are shown. (G and H) Quantification of intracellular trehalose from carbon-deprived wild-type and mutant M. smegmatis strains. The data in panel G are expressed as ratios of trehalose from acetamide-induced/acetamide-uninduced M. smegmatis expressing pYAB-EV and pYAB-Tre. Ratios are plotted from three independent experiments performed in triplicate. Representative data are shown for panel H. Error bars, standard deviations. The statistical significance of mutants versus the wild type (B and C) or with or without trehalase expression (G) was assessed by two-tailed Student *t* test. *, *P* < 0.05; **, *P* < 0.005. Download FIG S6, TIF file, 1.2 MB. Copyright © 2021 Pohane et al. 2021 Pohane et al. This content is distributed under the terms of the Creative Commons Attribution 4.0 International license.

How might trehalose recycling promote redox homeostasis under nutrient limitation? We noted that mycomembrane synthesis continues unabated in the Δ*sugC* mutant (see Fig. S4D) and that TMM remains at wild-type levels (Fig. 2F and G). The synthetic lethal interactions between *otsA* and *treYZ* or *lpqY*-*sugABC* in M. tuberculosis (64) suggest functional redundancy between the pathways encoded by these genes. The TreYZ pathway does not require energy to break down α-glucan into trehalose but OtsA and OtsB convert phosphorylated glucose intermediates to trehalose. In glucose-limited conditions, trehalose biosynthesis via the OtsAB pathway may also require additional ATP to drive gluconeogenesis. We considered whether induction of ATP-expensive trehalose anabolism might explain the oxidative stress that occurs in the absence of LpqY-SugABC.

Four lines of evidence support the first part of this model, e.g., that loss of recycling stimulates ATP-consuming trehalose biosynthesis. First, the M. smegmatis Δ*sugC* strain has lower ATP levels than the wild type (Fig. 3E). Second, we observed enhanced metabolism of fluorescently labeled glucose in the mutant (see Fig. S7). Third, while the expression of *otsA* did not change and the expression of one of the two M. smegmatis *otsB* homologs, (MSMEG_6043) was not detectable, the expression of the other *otsB* homolog, MSMEG_3954, was enhanced ∼4-fold in the absence of *sugC* (Fig. 4B). Finally, the levels of glucose-6-phosphate—the end product of gluconeogenesis—were elevated in the Δ*sugC* strain but suppressed in the Δ*otsA* strain (Fig. 4C), respectively, consistent with increased and decreased flux through this pathway.

10.1128/mBio.02801-20.7 FIG S7 Trehalose recycling limits glucose metabolism. (A) Fluorescent glucose analogue 2-NBDG was added to wild-type (WT), Δ*sugC*, and complemented (*C*Δ*sugC*) M. smegmatis strains that had been cultured in 0.02% glucose-supplemented medium. Samples were normalized by wet pellet weight. Aqueous extracts were processed using the same conditions and solvent systems used to detect cytoplasmic trehalose (see Fig. S6B, G, and H). Left TLC, fluorescent carbohydrates; right TLC, all carbohydrates. Boxed areas highlight prominent fluorescent species that were reliably enhanced in the Δ*sugC* mutant relative to wild-type or complement strains, and arrows denote the standards. STD, standard (purified 2-NBDG or trehalose). The experiment was performed three times; representative TLC results are shown. (B) Fluorescent TLCs were scanned and processed in ImageJ (99). The intensities of the most prominent fluorescent bands were normalized to the sum of all of the fluorescent bands. The data are plotted from three independent experiments. (C) Cartoon summary of Fig. S7A and B. In the absence of trehalose recycling, there is enhanced metabolism of 2-NBDG. Error bars, standard deviations. The statistical significance of the relative fluorescence intensity in wild-type versus Δ*sugC* strains was assessed by a two-tailed Student *t* test. *, *P* < 0.05. Download FIG S7, TIF file, 2.6 MB. Copyright © 2021 Pohane et al. 2021 Pohane et al. This content is distributed under the terms of the Creative Commons Attribution 4.0 International license.

We next tested the second part of our model, e.g., whether induction of trehalose anabolism upsets redox balance in carbon-deprived mycobacteria. Given the synthetic lethal interaction between *sugC* and *otsA* (64), we opted to deplete the trehalose pool by inducible trehalase overexpression. We compared the hydrogen peroxide sensitivity of strains that overexpress trehalase in wild-type, Δ*otsA*, and Δ*treYZ* backgrounds. Loss of OtsA, but not of TreYZ, rescued the sensitivity of M. smegmatis to hydrogen peroxide upon trehalase overexpression (Fig. 4D). These experiments indicate that trehalose replenishment by the OtsAB pathway can sensitize carbon-starved mycobacteria to ROS. Taken together, our data suggest that trehalose recycling limits energy consumption and oxidative stress during carbon limitation by alleviating the need for *de novo* biosynthesis.

### Trehalose recycling promotes *M. tuberculosis* survival in macrophages.

**FIG 5:**
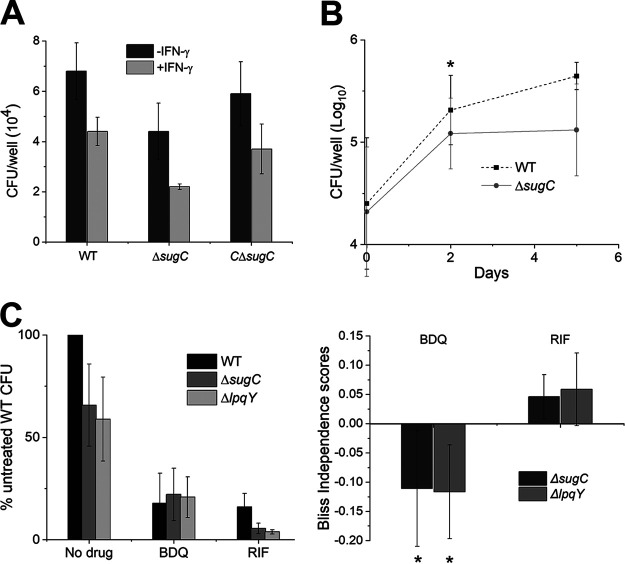
Trehalose recycling promotes M. tuberculosis survival in macrophages. (A) Survival of wild-type, Δ*sugC*, and complemented Δ*sugC* (CΔ*sugC*) M. tuberculosis strains in immortalized C57BL/6 bone marrow-derived macrophages (iBMDM) with or without IFN-γ treatment at 3 days postinfection. The experiment was performed at least three times in duplicate or triplicate; the results of one representative experiment are shown. (B) Wild-type and Δ*sugC* M. tuberculosis strain survival in IFN-γ-stimulated iBMDM at 0, 2, and 5 days postinfection. Log_10_-transformed data are combined from three to seven independent experiments performed in duplicate or triplicate. (C, left) Survival of wild-type, Δ*sugC*, and Δ*lpqY* M. tuberculosis strains in IFN-γ-activated iBMDM with or without bedaquiline (BDQ) or rifampin (RIF) at 2 days postinfection. The CFU from each condition were normalized to the untreated wild type. (Raw data are shown in Fig. S8B.) (Right) Bliss independence scores for mutant-drug interactions were obtained by subtracting the expected values for inhibition from the observed values. The expected values were calculated as described in Materials and Methods. Combined data from five (RIF) or six (BDQ) independent experiments are shown. Error bars, standard deviations. Statistical significance was assessed by a two-tailed Student *t* test on log_10_-transformed data at each time point (B) or by comparing expected and observed values for mutant-drug interactions (C, right). *, *P* < 0.05.

Deletion of *sugC* or *lpqY* inhibits M. tuberculosis replication in the acute phase of murine infection (20). Transposon insertions in *sugABC or lpqY* also attenuate pooled M. tuberculosis growth in interferon-gamma (IFN-γ)-activated or resting C57BL/6 bone marrow-derived macrophages (BMDM) (32). While it is likely that progressive carbon starvation underlies the *in vivo* and macrophage defects of trehalose recycling mutants, the precise mechanism(s) have not been clear. Our *in vitro* experiments support a model in which trehalose anabolism compensates for the loss of trehalose recycling but exacts energetic and redox costs. Since one consequence of IFN-γ activation is ROS production by the macrophage (65, 66), we first sought to test whether the magnitude of trehalose recycling mutant attenuation was different in the presence or absence of the cytokine. We confirmed that the M. tuberculosis Δ*sugC* mutant was defective for growing in immortalized BMDM and that this phenotype was reversed by genetic complementation (Fig. 5A and B). However, the IFN-γ-dependent decrease in the Δ*sugC* strain fitness relative to the wild type was very modest (see Fig. S8A), suggesting that sensitivity to ROS or to other, downstream stresses such as reactive nitrogen intermediates, acidic pH, and nutrient limitation (67, 68) does not fully account for attenuation in macrophages.

10.1128/mBio.02801-20.8 FIG S8 Survival of trehalose recycling mutant M. tuberculosis in macrophages with or without IFN-γ or with or without antibiotics. (A) Intracellular survival of the Δ*sugC* mutant in iBMDM relative to wild-type (WT) M. tuberculosis with or without IFN-γ at 3 days postinfection. CFU ratios were plotted from three independent experiments, including data from Fig. 5A. (B) Survival of wild-type, Δ*sugC*, and Δ*lpqY* M. tuberculosis strains in IFN-γ-activated iBMDM with or without bedaquiline (BDQ) or rifampicin (RIF) at 2 days postinfection. Log_10_-transformed data are combined from five or six independent experiments performed in duplicate or triplicate. Data normalized to the untreated wild-type strain are presented in Fig. 5C (left). Error bars, standard deviations. The statistical significance in assessed by a two-tailed Student *t* test by comparing the fold change in the CFU of the Δ*sugC* mutant relative to the wild type with or without IFN-γ activation (A) or by comparing log_10_-transformed data of mutants relative to the wild type for each condition. *, *P* < 0.05. Download FIG S8, TIF file, 0.3 MB. Copyright © 2021 Pohane et al. 2021 Pohane et al. This content is distributed under the terms of the Creative Commons Attribution 4.0 International license.

We next sought to determine whether dysfunctional energy metabolism compromises the fitness of trehalose recycling mutants during infection. To do this, we took a chemical-genetic epistasis approach. Bedaquiline inhibits ATP production by targeting the F_1_F_0_ ATP synthase (69, 70). Bedaquiline-treated M. tuberculosis is transiently able to maintain ATP levels by increasing oxidative and substrate-level phosphorylation (71, 72). Loss of trehalose recycling also results in ATP depletion (Fig. 3E) and enhanced respiration (Fig. 3D) *in vitro*. If these perturbations to (energy) metabolism are responsible for trehalose recycling mutant attenuation, we reasoned that bedaquiline should inhibit wild-type, Δ*lpqY*, and Δ*sugC* M. tuberculosis strains similarly, e.g., that the drug should not be additive with either of the mutations. Indeed, we found that the loss of *lpqY* or *sugC* was additive with treatment with rifampin, an antibiotic that does not impair mycobacterial energy metabolism (73, 74), but not with bedaquiline (Fig. 5C; see also Fig. S8B). Taken together, our data suggest that energy dysfunction that accompanies loss of trehalose recycling attenuates M. tuberculosis in macrophages.

## DISCUSSION

**FIG 6:**
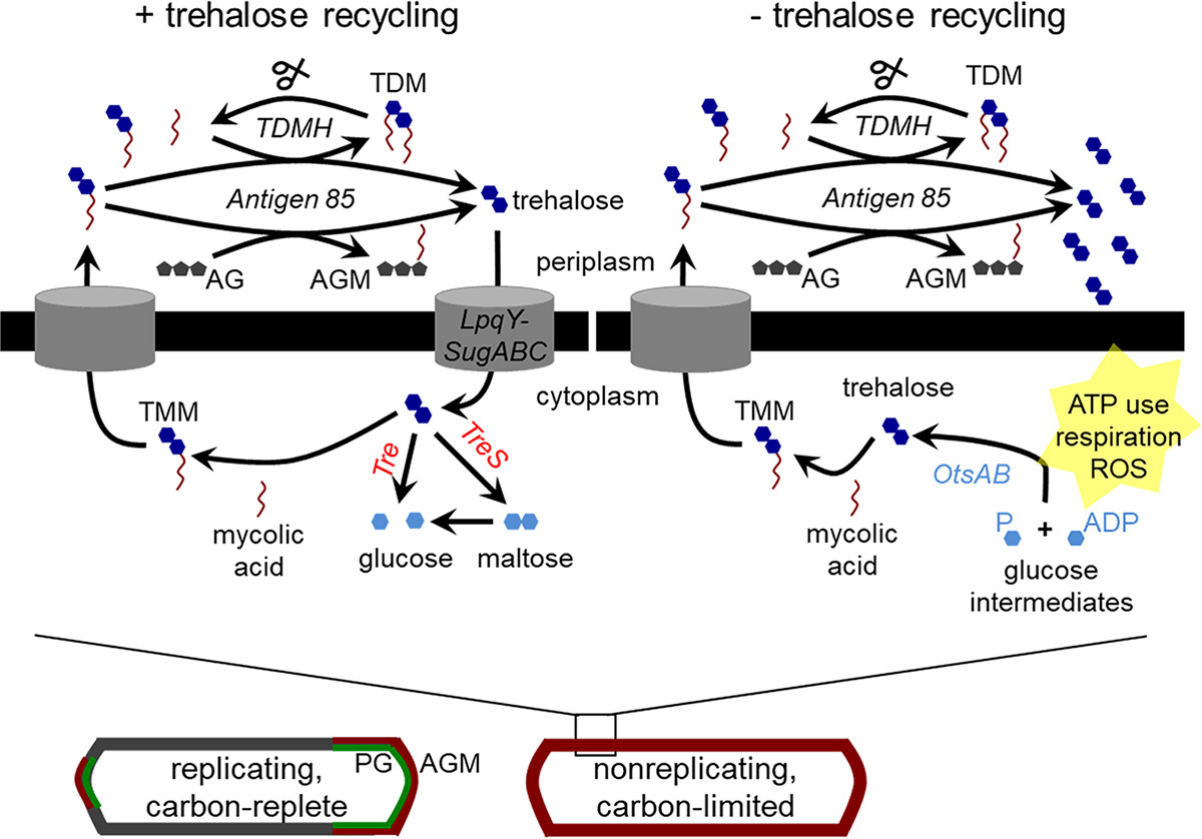
Model for the role of trehalose recycling in mycomembrane remodeling under nutrient or host stress. (Bottom left) Mycobacteria growing under carbon-replete conditions synthesize peptidoglycan (PG; green) and arabinogalactan mycolates (AGM; red) primarily at the poles of the cell. (Bottom right) Mycobacteria respond to growth-limiting carbon deprivation by turning over trehalose dimycolate (TDM) and synthesizing AGM along the entire cell periphery. Peptidoglycan metabolism, in contrast, is relatively inactive. (Top left) In carbon-deprived wild-type cells, the TMM building blocks are obtained at least in part from trehalose recycled by LpqY-SugABC. Trehalose may also be funneled to central carbon metabolism via TreS- or trehalase (Tre)-mediated catabolism. (Top right) In carbon-deprived mutants unable to recycle trehalose, TMM is supplied by *de novo* trehalose synthesis (dark arrow), which in turn depletes ATP, drives respiration, and confers ROS sensitivity.

Hints of mycomembrane plasticity began to appear in the early 1900s, when it was recognized that acid-fastness—a hallmark staining property still used for microscopy-based diagnosis of M. tuberculosis—varied with nutrient supply (75–77). More recent work supports the idea that the mycomembrane is reconfigured *in vivo* and in response to host-mimicking stresses (3, 5–13). The mechanisms by which these cell surface alterations occur are still emerging but have been attributed primarily to catabolic pathways (3, 6). We took advantage of recent advances in metabolic labeling (35, 78) to show that mycomembrane remodeling under *in vitro* carbon deprivation also involves anabolic reactions (Fig. 1C), a counterintuitive result since mycobacterial replication (see Fig. S1A) and presumably the overall metabolic activity are sluggish. Our data collectively indicate that the net result of such reactions is decreased TDM and spatial rearrangement of AGM (Fig. 6). We previously showed that synthesis of peptidoglycan along the nonexpanding sidewall of M. smegmatis is enhanced in response to cell wall damage (38). AGM synthesis under carbon starvation also occurs along the cell periphery (Fig. 1F), further supporting the notion that mycobacteria can edit their cell surface in a growth-independent fashion.

The adaptive consequences of mycomembrane remodeling are manifold (21, 79, 80). For example, bulk decreases in TDM and AGM abundance are known to increase mycobacterial cell permeability, which in turn enhances nutrient uptake and antimicrobial susceptibility (3, 4, 39). Although we do not observe gross changes in the amount of AGM under nutrient deprivation (Fig. 1D), the primary site of synthesis shifts from the pole to sidewall (Fig. 1F). The concomitant reduction in permeability (Fig. 1E)—despite an overall decrease in TDM abundance—suggests that the subcellular distribution of AGM also contributes to the barrier function of the mycobacterial cell envelope. Beyond enabling edits to the structural components of the mycomembrane, remodeling reactions liberate smaller molecules that influence cell physiology. Free trehalose released by TDM and AGM synthesis can be recycled into glycolysis or pentose phosphate intermediates or act as a stress protectant or compatible solute in the cytoplasm (6, 21–23). Our data suggest that it can also be directly refashioned into trehalose-containing, cell surface glycolipids (Fig. 2D and E), likely TMM. Free mycolic acids generated by TDM hydrolysis are components of biofilm matrix (7) and, like trehalose, serve as carbon sources (81). We speculate that they may additionally be reused together with recycled trehalose to make TMM.

How do mycobacteria power mycomembrane remodeling when faced with a loss of nutrients? The three isoforms of the TMM-consuming antigen 85 complex (Ag85C), encoded in M. tuberculosis by *fbpA*, *fbpB*, and *fbpC*, have partially redundant acceptor specificities (39, 82). However, only *fbpC* is upregulated in nutrient-starved M. tuberculosis (83, 84), making Ag85C an obvious candidate for performing synthetic reactions under that condition. Perhaps the more interesting question, however, is the source of the energetically expensive TMM building blocks. Breakdown of TDM by TDMH furnishes free mycolic acids and TMM, the latter of which could serve as a donor for sidewall AGM synthesis (7, 15). While such a pathway would not require ATP, it would be limited by the amount of TDM loss that can be tolerated without lysis (7, 34) or reduced resilience to host stress (3). Our data suggest that M. smegmatis and M. tuberculosis also generate TMM in the cytoplasm from recycled trehalose (Fig. 2D and E). An intracellular route of TMM generation would limit TDM loss, thereby preserving mycomembrane integrity. Use of recycled materials in turn would allow the mycobacterial cell to reap the benefits of sidewall AGM fortification while minimizing energy expenditure. In the absence of trehalose recycling, *de novo* synthesis supplies the sugar and mycomembrane remodeling continues unabated (see Fig. S4). The cost of from-scratch, OtsAB-mediated anabolism is not apparent under standard *in vitro* culture conditions but sensitizes M. smegmatis and M. tuberculosis to ROS (Fig. 3) and may contribute to defective M. tuberculosis growth during infection (Fig. 5) (20).

Trehalose is a cytoplasmic stress protectant and compatible solute and, in many types of bacteria, a carbon source (62, 85, 86). Mycobacteria and related organisms are relatively unique in using trehalose for extracellular purposes, to build their outer cell envelope. As the sugar fluxes in and out of central metabolism and the mycomembrane via several synthetic (OtsAB and TreYZ) and degradative (TreS and trehalase) processes, trehalose utilization may be particularly vulnerable to perturbations that induce redox and metabolic imbalances. Like carbon-limited Δ*sugC* M. smegmatis or M. tuberculosis strains, biofilm cultures of M. tuberculosis Δ*treS* have disruptions in energy and redox homeostasis (23). However, our data suggest that the mechanisms are distinct. In mature biofilms, trehalose is shunted away from TMM and TDM synthesis into glycolytic and pentose phosphate intermediates in a TreS-dependent manner (23). In contrast, we find that TMM levels are maintained during the time frame of our experiment, either by LpqY-SugABC, in wild-type organisms, or by *de novo* synthesis, in Δ*sugC* mutants (Fig. 6). While biofilm M. tuberculosis Δ*treS* mutants are likely more sensitive to ROS because they are depleted for the antioxidant precursor γ-glutamylcysteine (23), carbon-limited M. smegmatis Δ*sugC* mutants have higher levels of ROS-counteracting, cytoplasmic thiols (see Fig. S5C). Finally, biofilm M. tuberculosis Δ*treS* is hypersensitive to ATP-depleting bedaquiline (23), whereas intracellular Δ*sugC* and Δ*lpqY* mutants are more tolerant (Fig. 5C). These and other metabolite data are most consistent with the idea that enhanced ROS production and susceptibility (Fig. 3) in the absence of trehalose recycling stems from increased anabolism of the sugar rather than decreased catabolism. While we focus here on mycomembrane remodeling that occurs within 1 to 3 days of adaptation to carbon-limited medium, the TreS-dependent, trehalose-catalytic shift occurs in 4- to 5-week-old biofilms. Under our conditions, the loss of TreS has no impact on ROS susceptibility (see Fig. S6E). While we cannot rule out stress- or species-specific differences between the two studies, we favor a model in which the adaptive role of trehalose changes over time: early fortification of the cell envelope, to protect against immediate environmental insults, and later rewiring of central carbon metabolism, to maintain ATP and antioxidant levels. Trehalose recycling maintains redox and ATP homeostasis in the second case by driving glycolysis and the pentose phosphate pathway and in the first case by providing energetically inexpensive substrates for mycomembrane remodeling, thereby easing the demand for the products of these metabolic pathways.

The presence of a retrograde transporter enables trehalose to cycle in and out of the cell and serve as a metabolic node between the mycomembrane and cytoplasm. Recycling of the sugar is known to enhance M. tuberculosis survival in a mouse model of tuberculosis. It is widely hypothesized that the *in vivo* growth defects of trehalose recycling mutants stem from progressive carbon starvation (20, 21, 50). Nutrient deprivation coupled with loss of trehalose catabolism may indeed reduce fitness *in vivo*. However, our data suggest a more complex model, namely, that futile trehalose cycling consumes ATP and stimulates compensatory, ROS-generating respiration (Fig. 6). The energy and redox phenotypes of a trehalose recycling mutant resemble those elicited by other futile cycles (24–28) and some bactericidal antibiotics (29, 71, 72, 87, 88). Enhanced bacterial respiration has been proposed to increase drug efficacy (29, 30), and indeed, the loss of trehalose recycling sensitizes M. tuberculosis to multiple antibiotics (31). Here, we found that disrupted energy metabolism is the primary mechanism of attenuation for trehalose recycling mutant M. tuberculosis in macrophages (Fig. 5). Dysfunction triggered by forced *de novo* synthesis of energy-expensive macromolecules may be a fruitful avenue for potentiating both immune and antibiotic activity against bacterial pathogens, including those that inhabit growth-limiting, nutrient-deprived host niches.

## MATERIALS AND METHODS

### Bacterial strains and culture conditions.

M. smegmatis mc^2^155 was grown in Middlebrook 7H9 growth medium (HiMedia, India) supplemented with Tween 80 (7H9T) and glucose (2 or 0.02%) at 37°C unless otherwise specified in the text. Two-day-old primary cultures of M. smegmatis grown in 2% glucose were normalized to an optical density at 600 nm (OD_600_) of 0.1 in fresh 7H9T supplemented with 2 or 0.02% glucose and allowed to grow for 24 h. M. tuberculosis H37Rv strains (gifts from Rainier Kalscheuer) were grown in Middlebrook 7H9 medium (BD Difco, Franklin Lakes, NJ) supplemented with Tween 80 and OADC (BD BBL, Sparks, MD). For starvation of M. tuberculosis, cultures grown in 7H9T-OADC to an OD_600_ of 0.8 to 1.0 were collected by centrifugation and washed once with 7H9T (no OADC) and resuspended in 7H9T (starvation medium) to a normalized OD_600_ of 1. To prepare a strain that expresses *tre*, the gene that encodes trehalase, under an acetamide-inducible promoter, we PCR amplified *tre* from genomic DNA of M. smegmatis by using 4535For_Acet (TGATGTGCTCTAGAGTTCTGCAACAGACCGAGCC) and 4535Rev_Acet (GGCCTGATCTAGACATCGGGGCGTTCGCGG) primers. The resulting PCR product was ligated in pYAB033 vector (a gift from Yasu Morita) at the XbaI site and transformed in E. coli XL-1 Blue strain. The colonies were screened by colony PCR and the obtained plasmid was confirmed by sequencing. Bacteria used in this study are listed in Table 1.

**TABLE 1:** Strains used in this study

Strain	Source (references)
Immortalized C57BL/6 BMDM	Christopher Sassetti (93)
	
M. smegmatis	
mc^2^155	NC_008596 in GenBank (94)
Δ*sugC*	Rainer Kalscheuer (20)
Δ*sugC* pMV361-*sugC*	Ben Swarts (95, 96)
Δ*otsA*	Rainer Kalscheuer (51)
Δ*treYZ*	Rainer Kalscheuer (51)
Δ*treS*	Rainer Kalscheuer (51)
Δ*tre*	Rainer Kalscheuer (48)
Δ*otsA* pYAB*-tre*	This study
Δ*treYZ* pYAB*-tre*	This study
pYAB	Yasu Morita (97, 98)
pYAB-*tre*	This study
	
M. tuberculosis	
H37Rv	Rainer Kalscheuer (20)
Δ*sugC*	Rainer Kalscheuer (20)
Δ*lpqY*	Rainer Kalscheuer (20)
Δ*sugC* pMV306-*sugC*	Rainer Kalscheuer (20, 95)
	
E. coli XL-1 Blue	Agilent Technologies

### ROS sensitivity.

M. smegmatis grown in 0.02% glucose for 24 h were normalized to OD_600_ of 1. The cultures were then treated with 0.15% H_2_O_2_ for 10 min at 37°C with shaking. The trehalase overexpression strains were grown for 20 h in 0.02% glucose and then induced with 0.2% acetamide for an additional 10 h before being treated with 0.1% H_2_O_2_ for 10 min at 37°C with shaking. After H_2_O_2_ treatment, 3 μl of 10-fold serial dilutions made in phosphate-buffered saline (PBS) was spotted onto 7H9–2% glucose agar. For the thiourea rescue experiment, cultures were pretreated with 50 mM thiourea for 45 min prior to H_2_O_2_. For M. tuberculosis, cultures in starvation medium were grown for 5 days, normalized to an OD_600_ of 0.1 in fresh starvation medium, and then treated with 0.4% of H_2_O_2_ for 2 h at 37°C with shaking. After H_2_O_2_ treatment, 5 μl of 10-fold serial dilutions made in PBS were spotted on 7H10-OADC agar plate. For the vitamin C experiment, M. tuberculosis cultures in starvation medium were normalized to an OD_600_ of 0.1 in fresh starvation medium. The cultures were then treated with 20 mM vitamin C for 2 days. After vitamin C treatment, 5 μl of 10-fold serial dilutions made in PBS were spotted onto 7H10-OADC agar.

### Macrophage infections.

Immortalized C57BL/6 BMDM (iBMDM; a gift from Christopher Sassetti) were seeded at 10^5^ cells/well in 24-well tissue culture plate and incubated at 37°C overnight. M. tuberculosis was added at 5:1 multiplicity of infection (MOI; bacteria:iBMDM) and incubated for 4 h. After incubation, the coculture was washed twice with high-glucose Dulbecco modified Eagle medium (DMEM; Genesee Scientific, San Diego, CA) to remove extracellular M. tuberculosis, and fresh 5 mM DMEM-FBS-HEPES medium was added (fetal bovine serum [Genesee Scientific, San Diego, CA] and HEPES [Gibco, Paisley, PA]). IFN-γ (PeproTech, Rocky Hill, NJ) was added or not at 25 ng/ml concentration. For antibiotic susceptibility experiments, cocultures were treated or not with 5 µg/ml of bedaquiline (BDQ) or rifampin (RIF) for 2 days of the infection. The infected iBMDM were incubated for 0 to 5 days and then washed once with PBS and lysed with 0.05% Triton X-100 in PBS. After lysis, 10 or 50 μl of 10-fold serial dilutions made in PBS were respectively spotted or spread onto 7H10-OADC agar to determine the CFU.

### Bliss scoring.

Bliss interaction scores (89) for pairs of mutant-drug interactions were obtained by subtracting the expected values for inhibition from the observed values. The expected values were calculated using the formula E_M_ + E_A_ – E_M_E_A_, where E_M_ is the effect of the mutation (Δ*sugC* or Δ*lpqY*) and E_A_ is the effect of the antibiotic (BDQ or RIF). Statistically significant combinations that produced Bliss scores ≠ 0 were interpreted as nonadditive interactions.

### DHE staining.

M. smegmatis grown for 24 h in 7H9T–0.02% glucose was normalized to an OD_600_ of 1 with the same medium and then treated with 5 µM dihydroethidium (DHE; Sigma, St. Louis, MO) for 30 min at 37°C. Fluorescence was analyzed by flow cytometry.

### Total thiol abundance.

The protocol for measuring the total thiol content was adopted from (30). Briefly, 10 ml of M. smegmatis grown for 24 h in 7H9T–0.02% glucose was centrifuged at 2,500 × *g* for 5 min and washed with buffer containing 50 mM Tris-Cl (pH 8) and 5 mM EDTA, and the cell pellets were normalized by wet weight. Bacteria were resuspended in the same buffer and lysed by bead beating. Lysates were centrifuged at 16,000 × *g* for 15 min at 4°C, and 5,5′-dithiobis(2-nitrobenzoic acid) was added to 100 µl of supernatants to a final concentration of 0.05 mM. The total thiol content was estimated by determining the absorbance (λ) at 412 nm.

### Methylene blue.

M. smegmatis grown for 24 h in 7H9T–0.02% glucose was adjusted to an OD_600_ of 0.25. Cultures were split in two; one of these was treated with 0.005% methylene blue and aliquoted to a 96-well plate. The plate was sealed with Microseal B adhesive sealing films (Bio-Rad, UK) and incubated at 37°C for 4 h with shaking. The seal was then removed, and the absorbance (λ) at 665 nm was measured. The difference between the absorbance (λ) values at 665 nm for treated and untreated samples was plotted.

### ATP, glucose-6-phosphate, and NADP/NADPH quantitation.

The ATP concentration was measured by using a BacTiter-Glo (Promega, Madison, WI) luminescence kit. The glucose-6-phosphate (G6P) concentration and the NADP/NADPH ratio were respectively measured with an Amplite (AAT Bioquest, Sunnyvale, CA) colorimetric G6P assay and colorimetric NADP/NADPH ratio assay kits. M. smegmatis grown for 24 h in 7H9T–0.02% glucose was washed once with PBS. The pellets were resuspended in PBS and lysed by bead beating. Lysates were normalized by total protein concentration using a BCA protein assay kit (Pierce, Rockford, IL) and then processed according to the manufacturer’s protocol.

### Trehalose quantitation.

For intracellular trehalose detection, M. smegmatis grown for 24 h in 7H9T–0.02% glucose was washed once with PBS. Cell pellets were normalized by wet weight and then resuspended in chloroform-methanol (1:1) for overnight incubation with shaking. The suspension was centrifuged at 10,000 × *g* for 5 min, and the organic fraction was collected in a new tube. One part chloroform and one part water were added to the organic fraction and mixed vigorously in a shaker for 15 min. Suspensions were centrifuged, and the upper aqueous layers were processed according to the manufacturer’s instructions for the trehalose assay kit (Megazyme, Ireland). For extracellular trehalose detection, M. smegmatis were grown for 24 h in 7H9T supplemented with 2 or 0.02% glycerol. Cultures were normalized to an OD_600_ of 1 prior to centrifugation. The upper layer was collected and filtered through a 0.2-µm syringe. Filtrates were processed as described above to detect trehalose.

### Lipid extraction and TLC.

For extractable lipid analysis, 10 ml of culture was washed with PBS, and cell pellets were normalized by wet weight (M. smegmatis) or by OD_600_ (M. tuberculosis). To obtain TDM and TMM, cell pellets were extracted with chloroform-methanol (2:1). The extracted lipids were separated by thin-layer chromatography (HPTLC silica gel; Millipore, Billerica, MA) with chloroform-methanol-acetone (90:15:10) and chloroform-methenol-H_2_O (80:20:2) for TDM and TMM, respectively (35, 90). Then, 5% H_2_SO_4_ in ethanol was used to develop the TLC results. Covalent mycolate extraction was adopted an earlier study (91). Briefly, mycolic-arabinogalactan-peptidoglycan (mAGP) complex was extracted from 100 ml of culture as described previously (91). The pellet was resuspended in PBS and sonicated to lyse the cells. Lysates were centrifuged, and pellets were collected and washed with PBS. The pellets were resuspended in 2% sodium dodecyl sulfate (SDS) in PBS and incubated at 80°C for 3 h with intermediate shaking. They were then resuspended in 1% SDS, centrifuged, and washed twice with water, once with 80% acetone, and once with 100% acetone. The pellets were dried to obtain the final mAGP complex. The samples were normalized by mAGP weight and then resuspended in PBS plus 0.05% Tween 80 (PBST) by water bath sonication. To extract mycolic acids from mAGP, the suspension was treated with 5% tetrabutylammonium hydroxide (TBAH) overnight with shaking. The extracted mycolic acids were separated by treatment with an equal volume of dichloromethane, followed by treatment with an equal volume of 0.25 M HCl and washed with water as described previously (91). To extract free mycolic acids from culture supernatants, the OD_600_ of M. smegmatis grown for 24 h in 7H9T–2% or 0.02% glucose were normalized to 1 with 7H9T. The normalized cultures were centrifuged at 10,000 × *g* for 5 min and supernatants were collected and passed through a 0.25-µm syringe filter. Supernatants (1 ml) were treated with 5% TBAH for 1 h, followed by an equal amount of dichloromethane and overnight incubation at room temperature with shaking. The suspension was then centrifuged at 10,000 × *g*, and the lower organic layer was removed. The organic layer was evaporated, and the pellet was mixed with 40 µl of chloroform-methanol (2:1). Mycolic acids were separated by TLC using chloroform-methanol (96:4) as described previously (7). Next, 5% molybdophosphoric acid in ethanol was used to develop the TLC results.

### Fluorescent glucose labeling.

M. smegmatis cultured in 0.02% glucose-supplemented 7H9T was normalized to an OD_600_ of 1.0 in fresh medium and treated with a 5 µM concentration of the fluorescent glucose analogue 2-(*N*-(7-nitrobenz-2-oxa-1,3-diazol-4-yl)amino)-2-deoxyglucose (2-NBDG; Abcam, Cambridge, MA) for 2 h at 37°C with shaking. The cultures were centrifuged at room temperature for 5 min and 4,000 rpm and then washed twice with PBST. After normalizing to the wet weight, the pellets were extracted with chloroform-methanol (2:1) overnight. The organic extracts were separated from the cell suspension by centrifugation at room temperature for 15 min and 12,000 rpm and then treated with 1 volume of H_2_O for 15 min at room temperature. The aqueous and organic layers were separated from each other suspension by centrifugation at room temperature for 5 min at 12,000 rpm and then subjected to TLC using chloroform-methanol-H_2_O (80:20:2) and 1-propanol–ethyl acetate–water (6:1:3), respectively. The TLC fluorescence was recorded by the ImageQuant system (GE Healthcare) or developed using 5% H_2_SO_4_ in ethanol.

### Propidium iodide.

We assessed propidium iodide (PI) uptake as described previously (92). Briefly, 50 µg/ml PI was added to M. smegmatis that had been cultured in 0.02 or 2% glucose. After incubation for 15 min at 37°C, the samples were washed once with PBS, and the fluorescence was measured by flow cytometry.

### Cell envelope labeling.

Probes used in this study include alkDala (50 µM), HADA (500 µM), O-AlkTMM (50 µM), N-AlkTMM (250 µM), and 6-TreAz (50 µM). M. smegmatis labeling was performed mainly as described previously (38). Briefly, the OD_600_ was normalized to 1 in the same medium. Cultures were shaken in the presence of probes for 30 min at 37°C for M. smegmatis. After incubation, the cultures were washed twice with PBST and fixed or not fixed with 2% formaldehyde at room temperature for 10 min. After fixation, the cultures were washed with PBST. Alkynes were detected by CuAAC reaction with carboxyrhodamine-110 azide (Click Chemistry Tools, Scottsdale, AZ). Azides were detected on live, unfixed cells by SPAAC reaction with DBCO-Cy5 (Click Chemistry Tools). Finally, the cultures were washed three times with PBST, and the fluorescence was measured by flow cytometry. For M. tuberculosis, the OD_600_ values for carbon-starved and unstarved cultures were normalized to 1 in the same media. Cultures were shaken in the presence of probes for 3 h at 37°C and then washed twice with PBST and subjected to SPAAC overnight at 37°C. The cultures were washed three times with PBST and fixed with 4% formaldehyde overnight at room temperature prior to removal from the BSL3 facility.

### Microscopy analysis.

Fluorescence microscopy and image quantitation were performed exactly as described previously (38).

### qRT-PCR.

M. smegmatis was cultured in 0.02% glucose medium for 24 h. Cell pellets were resuspended in 1 ml of TRIzol reagent (Invitrogen, Carlsbad, CA) prior to bead-beating (MP Biochemicals lysing matrix B). After bead beating, 300 μl of chloroform was added to each tube. The tubes were centrifuged at 14,000 rpm for 15 min at 4°C. The upper aqueous layer was removed and resuspended in 600 μl of isopropanol in a fresh tube. The tube was kept at −20°C for 1 h to overnight and then centrifuged for 20 min at 4°C and 14,000 rpm to precipitate the RNA. The RNA-containing pellet was washed once with 75% ethanol by centrifugation for 5 min at 4°C and 14,000 rpm and then resuspended in RNase-free H_2_O. Next, 20 μg of RNA was treated with 2.5 μl of Turbo DNase (Ambion, Carlsbad, CA) in a final volume of 100 μl. The reaction mixture was incubated for 2 h at 37°C. The RNA was then cleaned up according to the manufacturer’s instructions for the RNeasy minikit (Qiagen). cDNA synthesis was carried out with 5 μg of the cleaned-up RNA according to the manufacturer’s instructions for SuperScript IV reverse transcriptase (Invitrogen). The cDNA was then used for qRT-PCRs (iTaq Universal SYBR green Supermix; Bio-Rad, Hercules, CA). We used the *sigA* gene as our internal control. The primers are listed in Table 2.

**TABLE 2:** Primers and sequences

Primer	Sequence
4535For_Acet	TGATGTGCTCTAGAGTTCTGCAACAGACCGAGCC
4535Rev_Acet	GGCCTGATCTAGACATCGGGGCGTTCGCGG
RT-otsA-For	ACTACACCAAGGGCATCGAC
RT-otsA-Rev	TCGCGATGTAGCTCTCGAC
RT-otsB-For (MSMEG_3954)	AACGAGAGCCTGGTCAATCT
RT-otsB-Rev (MSMEG_3954)	AGGGTCTGCTGGTAGGACTG
RT-otsB-For (MSMEG_6043)	GTGAGTCTTTCGGGGGATCT
RT-otsB-Rev (MSMEG_6043)	AATCGGATGTGACCAGCAG
RT-treY-For	CTCTCGACGTATCGGTTGC
RT-treY-Rev	AGGATGGGGGACAGATACAC
RT-treZ-For	CTCGACTACCTGGTCGATCTC
RT-treZ-Rev	ACCTCCGTAGGGTTCGTGTA
ForsigA	GGGCTACAAGTTCTCGACCT
RevsigA	CCGAGCTTGTTGATCACCTC
